# Nutrient Exposure Alters Microbial Composition, Structure, and Mercury Methylating Activity in Periphyton in a Contaminated Watershed

**DOI:** 10.3389/fmicb.2021.647861

**Published:** 2021-03-19

**Authors:** Alyssa A. Carrell, Grace E. Schwartz, Melissa A. Cregger, Caitlin M. Gionfriddo, Dwayne A. Elias, Regina L. Wilpiszeski, Dawn M. Klingeman, Ann M. Wymore, Katherine A. Muller, Scott C. Brooks

**Affiliations:** ^1^Oak Ridge National Laboratory, Biosciences Division, Oak Ridge, TN, United States; ^2^Oak Ridge National Laboratory, Environmental Science Division, Oak Ridge, TN, United States; ^3^Department of Chemistry, Wofford College, Spartanburg, SC, United States; ^4^Smithsonian Environmental Research Center, Edgewater, MD, United States; ^5^Pacific Northwest National Laboratory, Earth Systems Science Division, Richland, WA, United States

**Keywords:** periphyton, mercury, microbiome, methylmercury, nutrient addition

## Abstract

The conversion of mercury (Hg) to monomethylmercury (MMHg) is a critical area of concern in global Hg cycling. Periphyton biofilms may harbor significant amounts of MMHg but little is known about the Hg-methylating potential of the periphyton microbiome. Therefore, we used high-throughput amplicon sequencing of the 16S rRNA gene, ITS2 region, and Hg methylation gene pair (*hgcAB*) to characterize the archaea/bacteria, fungi, and Hg-methylating microorganisms in periphyton communities grown in a contaminated watershed in East Tennessee (United States). Furthermore, we examined how nutrient amendments (nitrate and/or phosphate) altered periphyton community structure and function. We found that bacterial/archaeal richness in experimental conditions decreased in summer and increased in autumn relative to control treatments, while fungal diversity generally increased in summer and decreased in autumn relative to control treatments. Interestingly, the Hg-methylating communities were dominated by Proteobacteria followed by Candidatus Atribacteria across both seasons. Surprisingly, Hg methylation potential correlated with numerous bacterial families that do not contain *hgcAB*, suggesting that the overall microbiome structure of periphyton communities influences rates of Hg transformation within these microbial mats. To further explore these complex community interactions, we performed a microbial network analysis and found that the nitrate-amended treatment resulted in the highest number of hub taxa that also corresponded with enhanced Hg methylation potential. This work provides insight into community interactions within the periphyton microbiome that may contribute to Hg cycling and will inform future research that will focus on establishing mixed microbial consortia to uncover mechanisms driving shifts in Hg cycling within periphyton habitats.

## Introduction

The conversion of mercury (Hg) to monomethylmercury (MMHg) is a critical area of concern in global Hg cycling. Once MMHg is produced, it is bioaccumulated in organisms and biomagnifies through food webs, ultimately impacting humans through the consumption of fish with elevated levels of MMHg. The transformation of inorganic Hg into MMHg is mediated by anaerobic microorganisms (Parks et al., 2013; Bravo and Cosio, 2020), predominantly from the Deltaproteobacteria, Methanomicrobia, and Firmicutes (Christensen et al., 2016, 2018). While significant work has examined Hg transformations in aquatic sediments (Bae et al., 2019; Branfireun et al., 2020; Xiang et al., 2020), less research has focused on aquatic microbial biofilms like periphyton. Recent work demonstrates that these biofilms may harbor significant amounts of MMHg (McDowell et al., 2020), but little is known about the community of microorganisms mediating Hg transformations.

Periphyton is a complex consortium of microorganisms including algae, fungi, bacteria, and archaea that are attached to inorganic and organic substrates in aquatic environments. Mercury methylation in periphyton has been shown in many different ecosystems and has been attributed to sulfate-reducing bacteria (SRB) and methanogenic archaea that occupy anoxic niches within the periphyton biofilm structure (Cleckner et al., 1999; Achá et al., 2011; Hamelin et al., 2011). To our knowledge, there is only one study that has identified Hg-methylating microorganisms in periphyton using the Hg methylation gene cluster, *hgcAB*, as a biomarker (Bae et al., 2019). Bae et al. (2019) found that periphyton and flocculant material from the Florida Everglades contained a diverse assemblage of *hgcAB*-containing microorganisms, including Deltaproteobacteria, Chloroflexi, Firmicutes, and Methanomicrobia. In addition to harboring Hg-methylating microorganisms, periphyton consortia can produce photosynthetic by-products and organic molecules that, in some instances, increase Hg bioavailability and MMHg production (Mangal et al., 2019a,b; Branfireun et al., 2020). Thus, overall MMHg production may be influenced by the interactions of the entire microbiome in the biofilm (Francoeur and Biggs, 2006; Lázaro et al., 2013, 2018).

Productivity of the periphyton microbiome is linked to abiotic factors such as temperature, light, stream velocity, and dissolved oxygen (Biggs et al., 1999; Hill et al., 2001; Beck et al., 2017). Nutrient types and concentration also play an important role in the periphytic algal composition and periphyton productivity (Hoellein et al., 2010). Often nitrogen and phosphorus are limiting nutrients for periphyton growth in lotic freshwater systems (Beck et al., 2017; Beck and Hall, 2018). Despite the role of periphyton in Hg cycling, the effect of nutrient addition on overall periphyton microbiome structure is not well characterized, and the impact of nutrient addition on the Hg-methylating microbial community is unknown. The single study of *hgcAB* in periphyton biofilms found that the abundance of *hgcAB* sequences was positively correlated with carbon and nutrient concentration and the overall abundances of sulfate-reducers and methanogens (Bae et al., 2019), indicating that the impact of nutrients on the methylating and overall periphyton microbial community could be a crucial determinant of MMHg production.

Therefore, the objective of this study was to quantify the impacts of nitrate and phosphate addition on the diversity, community composition, and Hg methylation activity of periphyton microbiomes in a Hg-contaminated stream across two seasons. Specifically, we hypothesized nutrient amendments would increase microbial interactions and diversity and therefore increase methylation potentials. To test this, nutrient diffusion substrates (NDS) were used to provide an artificial substrate for *in situ* periphyton growth and to test the response of the periphyton microbiome to nitrate and phosphate additions across two seasons.

## Materials and Methods

NDS cups were prepared to assess the impact of nitrate and phosphate exposure on periphyton microbial community composition and Hg methylation and MMHg demethylation. Periphyton biofilm growth, potential function, and community composition on the substrates were characterized with measurements of biofilm mass; chlorophyll *a*, *b*, and *c*; total ambient Hg and MMHg; Hg methylation and demethylation potential; and amplicon sequencing of the ITS2 region and 16S rRNA and *hgcAB* genes.

**Figure 1:**
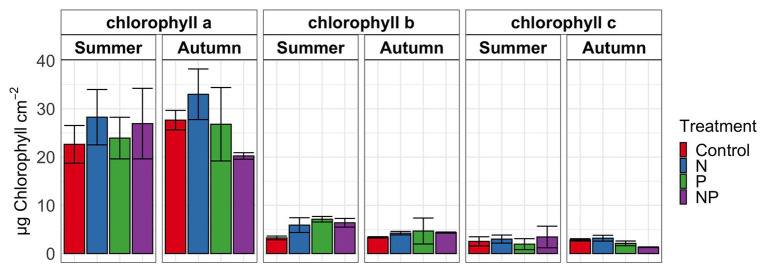
Chlorophyll *a*, *b*, and *c* in periphyton for summer and autumn experiments of control, nitrate (N), phosphate (P) and nitrate + phosphate (NP) nutrient exposures. Bars represent average of triplicates. Error bars represent standard deviation.

### Nutrient Diffusion Substrate Preparation

NDS cups (Costello et al., 2016) were constructed using black, 1-ounce plastic cups (#1 Poly-Cons®) with 2.5-cm holes drilled in the center of the lids (Supplementary Figure S1). The cups were filled to the top with a 2% agar solution (bacteriological grade, VWR; control and single nutrient treatments) or 3% agar solution (nitrate + phosphate). Four nutrient treatments were prepared: control, nitrate (0.5 M NaNO_3_), phosphate (0.5 M KH_2_PO_4_), and nitrate + phosphate (0.5 M NaNO_3_ + 0.5 M KH_2_PO_4_). For the nitrate treatment, the nitrate salt was dissolved in Milli-Q water, which was brought to boiling before adding the agar. The same method was attempted with the phosphate-containing treatments, but we found that the agar would not solidify when the phosphate salt was added prior to dissolving the agar. So, for the phosphate and nitrate + phosphate treatments, Milli-Q water was brought to a boil, the agar was added and allowed to dissolve, and then the nutrient salts were added to the hot agar solution and mixed well. This preparation method should not have resulted in any biocidal by-products as no reducing sugars were added (Tanaka et al., 2014). The hot agar was poured into the plastic cups and allowed to cool and solidify. The solidified agar was topped with a porous ceramic crucible cover disc (EA Consumables), and the cup cap was closed and sealed with hot glue. A total of 160 NDS cups were prepared for each deployment, 40 vials per treatment. After construction, NDS cups were stored in sealed plastic bags at 4°C until deployment (within 2 weeks of NDS construction). The NDS cups were attached in alternating order to 4 ft. angle iron bars using plastic zip ties and hot glue on the day of deployment (Supplementary Figure S2).

### NDS Deployment

The NDS cups were deployed in East Fork Poplar Creek (EFPC) in Oak Ridge, TN, United States. EFPC is contaminated with Hg and MMHg due to cold-war era industrial activities (Brooks and Southworth, 2011), and periphyton biofilms are known to be a major source of MMHg to the Creek (Olsen et al., 2016; Schwartz et al., 2019). EFPC has a high nutrient load and is mesotrophic to eutrophic along its length with respect to both nitrogen and phosphorus. EFPC km 13.8 was selected as the deployment site due to the relatively low nitrogen and phosphorus concentrations compared to other regions of the Creek (Avg. nitrate = 1.4 mg/l; Avg. phosphate = 0.18 mg/L). Two experiments were run, one from July 11, 2019 to August 12, 2019 (summer) and one from September 5, 2019 to October 4, 2019 (autumn). The average stream temperature for summer and autumn was 23.6 ± 1.14 and 22.9 ± 1.12°C, respectively. Numerous flood events occurred during the summer deployment while autumn did not have a flood event (Supplementary Figure S3). The 1-month incubation time was chosen to allow sufficient biofilm growth to develop the anaerobic micro-niches for Hg methylation (Olsen et al., 2016) but still have nutrient diffusion from the NDS cups (Supplementary Figure S4). After 1 month of incubation, the NDS cups were retrieved from EFPC, placed in plastic bins, covered in unfiltered EFPC water, and transported back to the laboratory (30-min holding time).

### Periphyton Functional Measurements

Periphyton measurements were normalized by disc area due to previously documented difficulties obtaining a precise biomass measurement (Beck and Hall, 2018).

Chlorophyll content was measured in each treatment (*n* = 3, per treatment) using a modified standard method (USA EPA, 1997). The periphyton discs were soaked in 15 ml of 90% acetone (v/v) for 2 h at room temperature, protected from light. The extraction solution was transferred to 15-ml centrifuge tubes and centrifuged for 15 min at 3,000 RPM. The absorbance of the supernatant solution was measured at 750, 664, 647, and 630 nm. The solution was acidified with 1 N HCl and absorbance was measured at 750 and 665 nm to determine pheo pigment-corrected chlorophyll *a*.

The ambient total Hg was extracted from the periphyton discs using an aqua regia digestion. Working in a fume hood, each disc was placed in an acid-clean glass jar with 10 ml concentrated, trace metal grade HCl and 3 ml concentrated, trace metal grade HNO_3_. The jars were loosely capped, and after 24 h, the aqua regia solution was carefully diluted for analysis with Milli-Q water and filtered through a 0.2-μm pore size polyethersulfone syringe filter. Total Hg extracted was quantified using Zeeman cold vapor absorbance spectrometry (LUMEX RA-915+, Ohio Lumex Co.). Briefly, SnCl_2_ [20% (w/v) SnCl_2_ in 10% (v/v) HCl] was added to an aliquot of sample to reduce the Hg to Hg(0). The sample was purged with ultra-high purity nitrogen, and the absorbance of the purged Hg(0) was measured. Triplicate discs of each treatment were digested and analyzed. The average detection limit was 24 pg.

Hg methylation and MMHg demethylation potential in the periphyton biofilms were assessed in the autumn experiment using methods described in Schwartz et al. (2019). Hg methylation and MMHg demethylation potential in the periphyton biofilms were assessed using stable isotopes purchased from Oak Ridge National Laboratory. Methylation was monitored by the formation of MM^201^Hg from ^201^Hg (96.17% purity) while demethylation was monitored by the loss of MM^202^Hg. The MM^202^Hg was synthesized in-house from ^202^Hg (95.86% purity) using the cobalamin method (Bancon-Montigny et al., 2004). For the methylation/demethylation assay, surface water (20 ml) from the NDS deployment site in EFPC was added to 60-ml, clear glass jars with PTFE-lined caps. The isotopes were spiked into the surface water (1.8 μg ^201^HgCl_2_ and 1.05 ng MM^202^HgCl), and the spiked solution was gently mixed by swirling and then allowed to equilibrate at room temperature (~20°C) under ambient laboratory lighting for 1 h. After 1 h, one periphyton disc was carefully added to each jar, biofilm side down. The jars were tightly capped, flipped, and incubated lid side down on the lab bench, underneath an aquarium light. For each of the four nutrient treatments (control, nitrate, phosphate, and nitrate + phosphate), triplicate jars were sacrificially sampled at 0, 24, 48, and 72 h. Surface water controls (unfiltered EFPC surface water only) were established and sampled at 0 and 72 h. At each timepoint, the incubation microcosms were acidified with 10 ml of 18% (w/v) KBr/5% (v/v) H_2_SO_4_ and 2 ml of 1 M CuSO_4_ and the isotope dilution spike for MMHg analysis was added to each microcosm. The preserved microcosm samples were stored at 4°C until extraction and distillation for MMHg analysis. The periphyton microcosms were prepared for MMHg analysis *via* total digestion and extraction following the Bloom et al. method (Bloom et al., 1997). The extracted sample was then distilled following EPA Method 1630 (USA EPA, 2001) and analyzed *via* Isotope Dilution-Gas Chromatography-Inductively Coupled-Mass Spectrometry (ID-GC-ICP-MS). A sediment standard reference material (ERM-CC580) was prepared with each batch of samples. MMHg recovery for the SRM averaged 90.3% ± 5.3. The detection limits for each MMHg isotope averaged ranged between 1.8 and 12.2 pg.

Methylation rate potentials were estimated using the transient availability kinetic model described by Olsen et al. (2018). The transient availability model combines kinetic expressions for multisite sorption of Hg and MMHg, Hg (II) reduction/Hg (0) oxidation, and methylation/demethylation kinetics. The ^201^Hg and MM^202^Hg time course data from the methylation/demethylation assay were fit with the transient availability model, and rate coefficients for the other kinetic reactions in the model were taken from Olsen et al. (2018). Herein, methylation and demethylation potentials refer to the fitted rate constant from the transient availability model results.

### Periphyton Microbiome Characterization

To isolate microbial DNA, samples were first sonicated for 10 min before extraction with the DNeasy PowerMax kit (Qiagen, Hilden, Germany). After extraction, a two-step PCR approach was used with barcode tagged templates and primers targeting the V4 region of the 16S rRNA gene for archaea and bacteria and the ITS2 region of the ribosomal operon for fungi using pooled primer sets (Supplementary Table S1) to increase coverage of archaeal, bacterial, and fungal marker genes (Cregger et al., 2018). Hg methylation genes (*hgcAB*) were amplified with a two-step PCR following methods and primers (Supplementary Table S1) previously described (Gionfriddo et al., 2020). After PCRs, all experimental units were pooled equally and purified with Agencourt AMPure XP beads (0.7:1 bead-to-DNA ratio; Beckman Coulter Inc., Pasadena, CA, United States). Negative controls with extraction blanks and no template controls were added to the pool before we performed Illumina MiSeq paired-end sequencing using a 9 pM amplicon concentration with a 15% PhiX spike (2 × 300 cycles). Amplicon sequences have been deposited in the BioProject database under the accession PRJNA688286.^1^

Microbial sequences (16S, ITS2) were processed with the QIIME 2 v 2019.10 platform (Bolyen et al., 2019). Paired sequences were demultiplexed with the plugin demux and quality filtered (denoised, dereplicated, chimera filtered, and pair-end merged) and processed into Sequence Variants (SVs) with the dada2 plugin (Callahan et al., 2016). Taxonomy was assigned using a pre-trained Naive Bayes classifier based on SILVA trimmed to the 515F/806R primer pair (16S), or the Unite (ITS) databases and unassigned sequences were removed. *hgcAB* sequences were filtered and trimmed to 201-nt base pairs using Trimmomatic (Bolger et al., 2014) and dereplicated, and singletons and chimeras were removed with VSEARCH (Rognes et al., 2016). SVs were generated with dada2 and taxonomy was assigned using the reference package ORNL_HgcA_201.refpkg (Gionfriddo et al., 2020).

### Statistical Analysis

All statistical analyses were performed in R with phyloseq and visualized with ggplot2. An ANOVA and Tukey HSD were used to test differences of chlorophyll *a*, chlorophyll *b*, chlorophyll *c*, MMHg demethylation, and Hg methylation rates with season and nutrient treatment as factors. Pearson correlation was used to test the correlation of SV richness and environmental measurements (mercury, chlorophyll *a*, chlorophyll *b*, chlorophyll *c*, methylation, and demethylation rates). Environmental measurement correlations with microbiome taxa were assessed with the Pearson correlation test, with *p* values corrected for multiple comparisons by the false-discovery rate method. For alpha diversity, Hill numbers (or effective numbers of species) were calculated with the package hillR to control the contribution of rare taxa to the diversity metrics. In general, the diversity measures are weighted differently by the diversity order (Dx), which calculates Hill numbers weighted differently by species abundance distributions (Hill, 1973; Chao et al., 2012). For example, at D_0_, all species are equally weighted, at D_1_, species are proportionally weighted to relative abundance, and at D_2_, rare species are down-weighted. An ANOVA was performed to test the effect of nutrient treatment and/or season on Hill numbers. Beta diversity was visualized with non-metric multidimensional scaling ordinate (NMDS) based on Bray-Curtis distances. A perMANOVA was performed to test the role of nutrient treatment on overall community structure.

### Cross-Domain Association Networks

The R package Spiec-Easi (Sparce InversE Covariance Estimation for Ecological Association Inference) was used to construct cross-domain microbial networks at the SV level for archaea, bacteria, and fungi. SV tables for each nutrient addition were filtered to include taxa present in 20% of samples. The sparsity parameter *λ* was set to 10 with the minimum *λ* of 0.001 and model properties were calculated with the igraph package. Hub taxa were identified as those above the 90th percentile of network SVs for the measures of degree and betweenness centrality as outlined in Agler et al. (2016).

## Results

### Periphyton Functional Measurements

**Figure 2:**
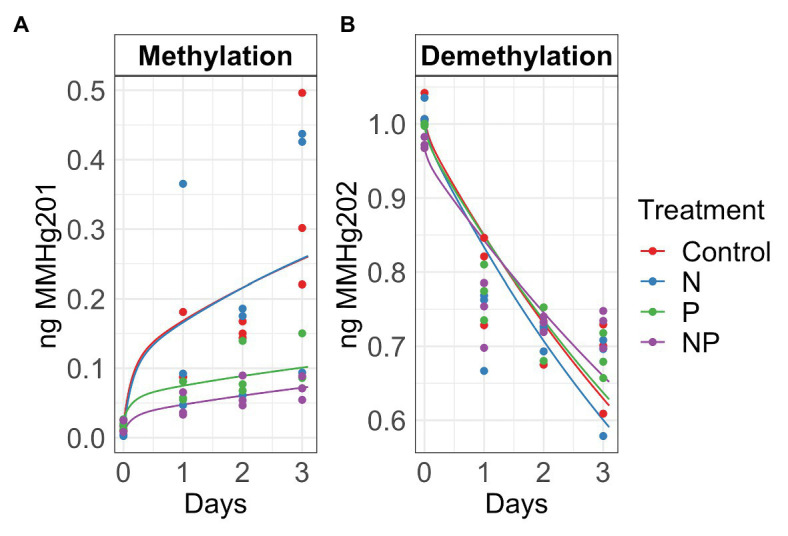
Autumn methylation **(A)** and demethylation **(B)** rates calculated with the Transient Availability Kinetic Model of control, nitrate (N), phosphate (P), and nitrate + phosphate (NP) nutrient exposures. The initial ^201^HgCl_2_ and MM^202^HgCl spikes were 1.8 *μ*g and 1.05 ng, respectively.

Summer and autumn samples had more chlorophyll *a* than chlorophyll *b* (*p* < 0.001). Chlorophyll *b* varied by treatment and season (*p* = 0.03) with the higher values measured in nutrient treatments in summer (Figure 1). Nitrate and nitrate + phosphate treatments did not differ in chlorophyll *a* content in summer, but nitrate had higher content of chlorophyll *a* that nitrate + phosphate exposures in autumn. Ambient Hg was higher in control and nitrate-exposed samples than phosphate and nitrate + phosphate samples (Supplementary Figure S5). Control and nitrate treatments had higher ambient MMHg levels in the biofilms than phosphate (*p* < 0.05) and nitrate + phosphate (*p* < 0.02) treatments (Supplementary Figure S6). Methylation and demethylation rate potentials were measured in the autumn experiment. The production of MMHg was highest in treatments without phosphate (*p* > 0.05) while demethylation rate potentials did not vary by treatment (*p* = 0.98; Figure 2). Methylation in the surface water controls was small (0.03 ng over 72 h) compared to the treatments, but demethylation in the surface water control was substantial (21% of the initial amount over 72 h compared to 27–34% of the initial amount for the treatments).

### Archaeal/Bacterial Community

**Figure 3:**
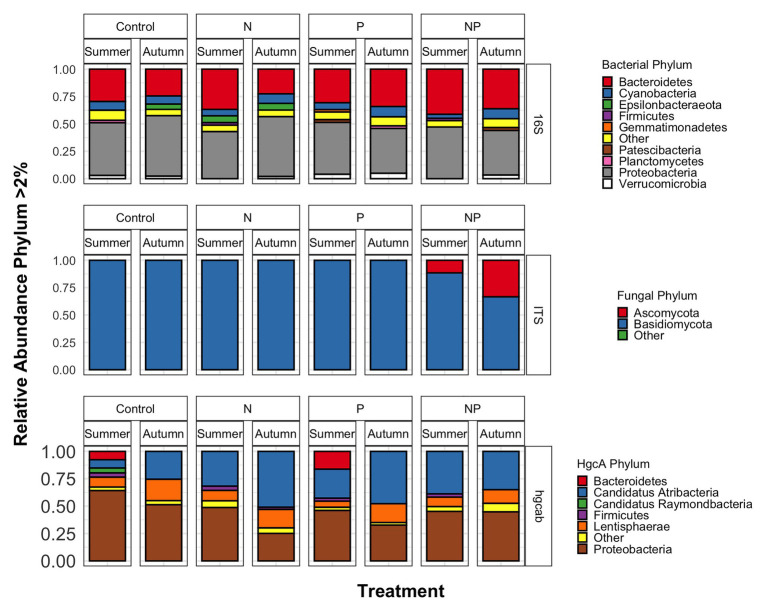
Phyla relative abundances of bacterial/archaeal (16S rRNA), fungal (ITS), and mercury methylating (*hgcAB*) microbial communities of control, nitrate (N), phosphate (P), and nitrate + phosphate (NP) nutrient exposures in summer and autumn experiments.

**Figure 4:**
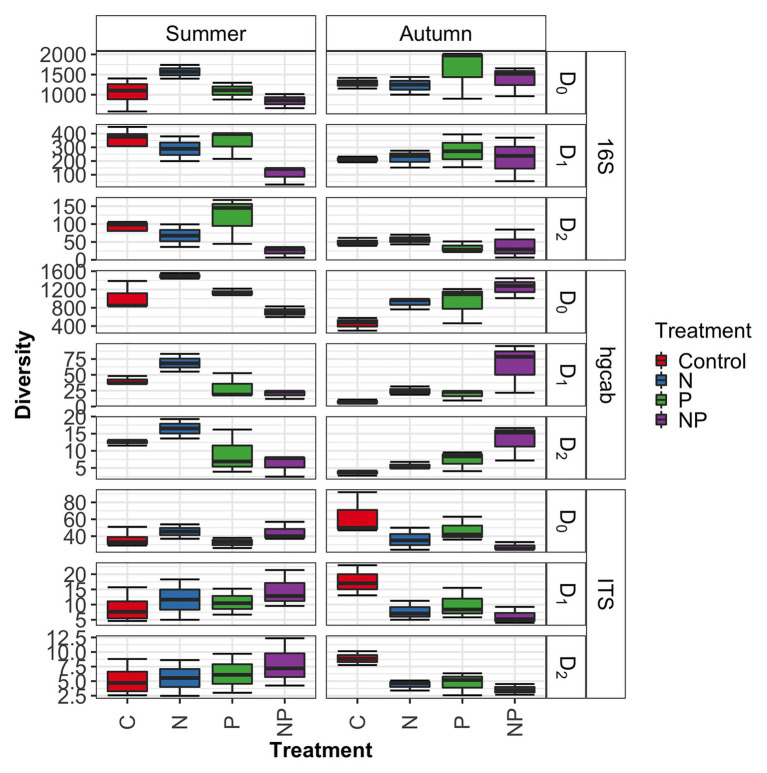
Microbiome diversity across control, nitrate (N), phosphate (P), and nitrate + phosphate (NP) nutrient exposure treatments and summer and autumn seasons measured by Hill’s numbers.

**Figure 5:**
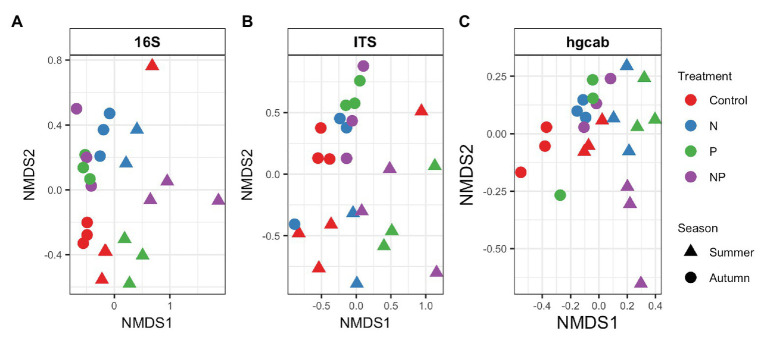
NMDS of Bray-Curtis dissimilarity of **(A)** bacteria/archaea (16S rRNA), **(B)** fungi (ITS), and **(C)** mercury methylating (*hgcAB*) periphyton communities of control, nitrate (N), phosphate (P), and nitrate + phosphate (NP) nutrient exposures in summer and autumn experiments. Color represents treatment while shape indicates the season periphyton was sampled.

Bacterial phyla Proteobacteria (43–55%) and Bacteroidetes (25–42%) dominated 16S rRNA datasets in all samples across all treatments followed by Cyanobacteria (4–9%; Figure 3). Bacterial diversity differed by treatment (*p* = 0.03) at only D_0_. D_0_ does not weight diversity while D_1_ weights diversity by relative abundance and D_2_ down-weights rare or low abundant taxa, suggesting that diversity changes were changes in number of SVs not specific to rare or dominant members (Figure 4). Community composition measured by Bray-Curtis distances of all bacterial and archaeal members in the microbiome was influenced by both season and nutrient amendment (*p* = 0.001; Figure 5A).

Numerous bacterial families correlated with chlorophyll *a* or chlorophyll *b* measurements. Bacterial families Shewanellaceae, Methylophilaceae, Arcobacteraceae, Acetobacteraceae, Pseudomadaceae, and Peptococcaceae positively correlated with chlorophyll *a* (Supplementary Table S3). Burkholderiaceae, Devosiaceae, Flavobacteriaceae, Micropepsaceae, Rhizobiaceae, and Rhodanobacteraceae positively correlated with chlorophyll *b* (*p* < 0.05). Cyanobacteria phylum did not correlate with methylation (*p* = 0.56) or demethylation (*p* = 0.89) potentials. However, cyanobacterial families Cyanobacteriaceae (0.02), Microcystaceae (*p* = 0.03), and an uncultured cyanobacterium (*p* = 0.006) negatively correlated with methylation potential while *Arcobacteraceae* positively correlated with methylation potential (*p* = 0.02). In addition to Arcobacteraceae, other bacterial families had a positive relationship with mercury methylation such as Desulfovibrionaceae, Eubacteriaceae, Dysgonomonadaceae, Marinilabiliaceae, Paludibacteraceae, Methylophilaceae, Cyclobacteriaceae, Rikenellaceae, and Rhodocyclaceae (*p* < 0.05; Supplementary Table S4). The bacterial families Desulfovibrionaceae, Burkholderiaceae, Pseudomonadaceae, Shewanellaceae, and Arcobacteraceae had a positive relationship with MMHg demethylation potential (*p* < 0.05; Supplementary Table S4).

### Fungal Community

Basidiomycota (69–99%) dominated samples across all treatments and nitrate + phosphate-treated samples were enriched with Ascomycota in both seasons (5–10%; Figure 3). Fungal diversity varied by season and treatment for D_0_ (*p* = 0.03) and D_1_ (*p* = 0.04). For both D_0_ and D_1_, fungal diversity increased with all treatments in the summer and decreased in diversity in the autumn when compared to the control treatment (Figure 4). Fungal differences were not detected for D2 (*p* > 0.05; a metric that down-weights rare taxa), suggesting that the increase in summer and decrease in autumn diversity were due to changes in rare taxa abundances. Fungal community composition varied by nutrient amendment and season (*p* = 0.006; Figure 5B). Fungal diversity did not correlate with chlorophyll measurements, Hg methylation, or demethylation potential (*p* > 0.25; Supplementary Table S2). The only fungal family with a positive relationship with chlorophyll *b* was *Pleasporaceae* (*r* = 0.48, *p* = 0.05).

### Mercury Methylating Community

The potential Hg-methylating bacterial community was dominated by Proteobacteria (25–55%) and Candidatus Atribacteria (23–50%; Figure 3). All nutrient addition treatments increased the relative abundance of Desulfovibrionales (5% up to 20%) and decreased relative abundance (40% down to 5–10%) of Desulfuromonadales compared to control samples. Diversity of *hgcAB* varied by nutrient addition treatment and sampling time for Hill numbers at D_0_ (*p* = 0.001), D_1_ (*p* = 0.001), and D_2_ (*p* = 0.002). Diversity in summer was highest for nitrate-amended samples (*p* < 0.05) while NP and P amended were lower than control (Figure 4). In autumn, however, nitrate + phosphate contained the highest diversity of Hg-methylating bacteria followed by nitrate and phosphate exposures. Hg methylation potential was negatively correlated with methylator diversity (*r* = −0.58, *p* = 0.04) in autumn. The Hg-methylating bacterial community was structured by sampling month as well as nutrient amendment (*p* = 0.003, Figure 5C). Desulfarculaceae and chlorophyll *a* had a positive relationship (*p* = 0.001) while Geobacteraceae and Methanoregulaceae (*p* < 0.05) had a positive relationship with chlorophyll *b*. Desulfobacteraceae and chlorophyll *c* had a positive relationship (*p* = 0.05; Supplementary Table S5). Desulfovibrionaceae had a positive relationship with Hg methylation potential and a negative relationship with Hg demethylation potential (Supplementary Table S6).

### Cross-Domain Correlations

**Figure 6:**
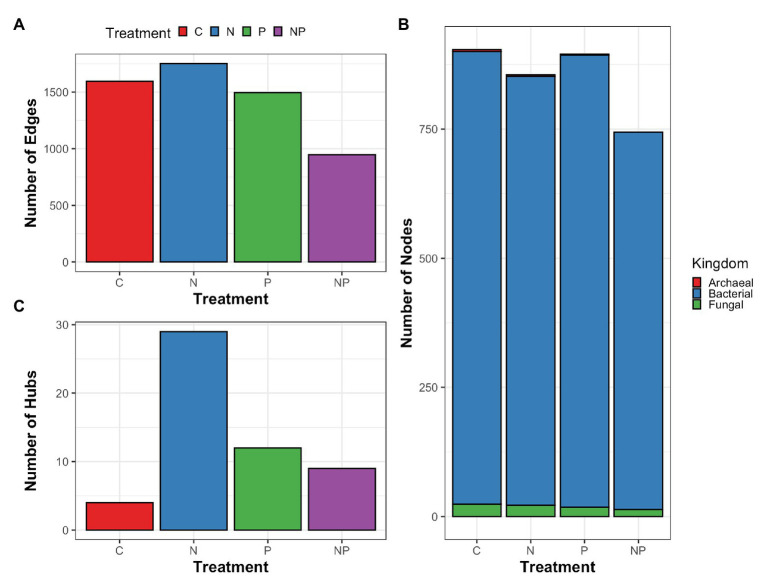
Summary of cross-domain correlation networks of periphyton archaea, bacteria, and fungal associations (edges; **A**) between microorganisms (nodes; **B**) as well as taxa with significantly higher interactions than other nodes (hubs; **C**) for control (C), nitrate (N), phosphate (P), and nitrate + phosphate (NP) nutrient exposure experiments.

To explore how microorganisms interacted within the periphyton communities rather than at the individual relative abundance level and how the interactions responded to nutrient amendments, we constructed microbial networks for each nutrient amendment. Microbial networks contain two components: nodes and edges. Nodes represent microorganisms and edges represent the associations between the nodes. A taxa hub is a node that is significantly associated with more edges and thus considered more connected within the network compared to other nodes. We found that across nutrient treatments, microbial networks differed in number of edges and hubs (Figure 6). The nitrate treatment and phosphate treatment networks had the highest number of edges while the nitrate + phosphate network was the sparsest in terms of edges. Bacterial taxa accounted for 730–876 nodes and fungal taxa accounted for 14–24 nodes in microbial networks of all nutrient amendments (Supplementary Figure S7). Control, nitrate, and phosphate networks contained 2–4 archaeal nodes while the nitrate + phosphate network did not contain archaeal nodes. The highest number of taxa hubs was determined in the nitrate amendment network with 29 taxa hubs followed by phosphate and nitrate + phosphate microbial networks with 12 and 9 taxa hubs, respectively. The control sample network contained only 4 taxa hubs. All taxa hubs across nutrient amendments were dominated by bacterial phyla with fungi only accounting for a single hub that was in the nitrate amendment and no archaeal hubs were detected (Supplementary Table S7). Overall, microbial networks had a diverse mix of microorganisms but were dominated by the bacterial phyla Proteobacteria and Bacteroidetes with Basidiomycota dominating the fungal phyla.

Most hubs were specific to nutrient amendments except for the bacterial genera *Azospira*, *Sphingopyxis*, and *WCHB1-32* that were found in the microbial networks corresponding to nitrate and phosphate nutrient amendments. Hubs from the control network comprised *Desulfomicrobium*, *Erythrobacter*, and *Lacibacter*, all of which were not detected hubs in other nutrient-amended networks. The top hub for the nitrate network was *Clostridium* followed by *Hydrogenophaga* and *Stenotrophomonas*.

## Discussion

Periphyton biofilms have received recent attention for their role in Hg cycling, but little is known about the structure or community interactions of this complex microbiome. The periphyton microbiome is diverse and composed of complex cross-kingdom interactions. In this study, we assessed the impact of nutrient amendments on the periphyton microbiome across two seasons. We detected differences in the periphyton microbiome in terms of diversity, composition, community structure, and Hg methylation across all treatments. Such characterizations are important as it is well known that microbiome function is not dependent on only one microorganism but rather the community as a whole.

Periphyton algal communities displayed sensitivity to season as well as nutrient addition. From summer to autumn, we found a significant decrease in chlorophyll *b* relative to the previous season suggesting a change in the abundance of green algae. This pattern may be due to variations in physiochemical parameters such as nutrients, light, and temperature. Temperature is a large determinant of algal growth (Allen and Hershey, 1996; Teubner, 2000; Pfeiffer et al., 2013; Yadav et al., 2018) but the water temperature in our experiment did not change. Additionally, our nutrient exposure was consistent and regulated by the NDS cups across seasons. Algal community differences across season may also be due to different disturbance events. Summer samples had numerous flood events that likely scoured the periphyton biofilms to varying extents and required re-establishment and regrowth (Francoeur and Biggs, 2006) while the autumn samples had no flood events. Nonetheless, the algal community changed with nutrient amendments differently across season. The highest algae and cyanobacteria abundance were measured in nitrate treatments while nitrate + phosphate treatments had the lowest abundance in autumn, suggesting that algal communities may be nitrogen and not phosphorus limited in autumn. Periphytic nitrogen limitation in aquatic systems has been confirmed (Dodds and Welch, 2000; Yadav et al., 2018); however, phosphorus limitation is common (McDowell et al., 2020). It is possible that the associated changes with phosphorus may be directly due to the interaction of nutrient status with light availability and temperature (McCall et al., 2017) or indirectly from changes in secondary metabolites (Leflaive and Ten-Hage, 2007) or negative interactions from microbiome changes associated with physiochemical properties (Beck and Hall, 2018). Hence, the driving mechanisms causing changes in algal abundances associated with phosphate addition need to be further examined.

The composition and diversity of bacteria, archaea, and fungi responded differently to nutrient amendments and season. Archaeal/bacterial and fungal richness varied across nutrient amendments. Interestingly, alterations in fungal diversity were driven by loss of rare taxa within the biofilm. Diversity changes were not associated with changes in algal abundances though specific bacterial and fungal families’ relative abundances demonstrated a relationship with algal abundance changes. The relative abundance of *Acetobacteraceae*, *Methylophilaceae*, *Pseudomadaceae*, *Rhizobiaceae*, and *Rhodanobacteraceae* demonstrated a relationship with algal community abundances. The bacterial family Acetobacteraceae are capable of acetic acid oxidation (Kersters et al., 2006) while Methylophilaceae, Pseudomadaceae, Rhizobiaceae, and Rhodanobacteraceae (Hanson and Hanson, 1996) oxidize methanol. Methanol and other C1 compounds are typical by-products of algal growth (Nesvizhskii and Aebersold, 2005; Taubert et al., 2019), suggesting that the relationship between these bacterial families and algae is from differences in substrate utilization.

Previous research has suggested that adding general cyanobacteria to periphyton microcosms enhanced MMHg production (Lázaro et al., 2019). Here, we found that the cyanobacteria relative abundance at the phylum level did not correlate with methylation rates, but we found that the cyanobacteria family Arcobacteraceae increased in relative abundance as Hg methylation rates increased. Interestingly, the cyanobacteria families Cyanobacteriaceae and Microcystaceae were negatively correlated with Hg methylation rates. This suggests competitive interactions within periphyton biofilms across cyanobacterial species that may have distinct implications for Hg cycling within these communities.

In addition to a diverse overall microbiome, periphyton contained a diversity of potential Hg-methylating organisms. These hgcA-containing microorganisms were dominated by Proteobacteria followed by Candidatus Atribacteria. The bacterial phylum Proteobacteria has previously been found to dominate periphyton (Bae et al., 2019) but not Atribacteria. Sediment samples collected from the same study site (EFPC) also found an abundance of Proteobacteria, but rather than Candidatus Atribacteria, the sediment community had an abundance of Nitrospirae and Chloroflexi that dominated the Hg-methylating community (Gionfriddo et al., 2020) Candidatus Atribacteria are a ubiquitous and difficult to cultivate phylum (Katayama et al., 2020). Given the vast interaction networks the periphyton microbiome demonstrated, networks of the periphyton may aid in future isolation endeavors of these taxa as well as other difficult-to-cultivate microorganisms.

Recent work in pure culture experiments (Yu et al., 2018) and peatland field incubations (Hu et al., 2019) have shown that syntrophy can be important for Hg methylation and microbial community analysis in the Florida Everglades has suggested this in periphyton (Bae et al., 2019). Our microbial community contained orders commonly associated with Hg methylation such as Desulfovibrionales, Desulfuromonadales, and Clostridiales (Gilmour et al., 2013; Jones et al., 2020; Peterson et al., 2020), but Hg methylation also correlated with numerous bacterial families not containing *hgcAB* such as the propionate-producing bacterial family Paludibacteraceae (Ueki et al., 2006) and the bacterial family Rikenellaceae that contains butyrate-producing genera (Revsbech et al., 1983). Butyrate and propionate syntrophs and SRB are hypothesized to enhance methylation in environments lacking sulfate or where other energy sources are limiting (Bravo and Cosio, 2020). Additionally, we found that *Desulfomicrobium* and *Erythrobacter* were important hubs in our control sample network. *Desulfomicrobium*, a SRB capable of mercury methylation (Gilmour et al., 2013), interacted directly with seven taxa in the periphyton microbiome. *Erythrobacter* are photosynthetic (Koblížek et al., 2003) and have been previously reported to play a crucial role in carbon and energy metabolism (Kolber, 2001; Teramoto et al., 2010; Hu et al., 2012). This suggests that not only the Hg methylators themselves, but also the supporting and interacting bacterial communities of the periphyton complex may be important in Hg methylation processes and merits future exploration.

Microbial network analysis identified potential community interaction changes and microbial hub taxa that differed across nutrient amendments. Hub taxa are taxa with more connections within the network and thus considered to interact with a larger number of other taxa within the community. The abundance of microbial hubs has been shown to correlate to the abundance of important functional attributes of nutrient cycling (Faust et al., 2015; Toju et al., 2018; Shi et al., 2020). Indeed, nitrate amendments resulted in the highest number of hubs as well as enhanced Hg methylation potential. Conversely, phosphate and nitrate + phosphate networks increased in hubs relative to control treatments but had the lowest Hg methylation potentials. Despite an increase in hub taxa, phosphate and nitrate + phosphate networks had a lower number of edges compared to control samples. This suggests that while a few taxa increased the number of interactions in the periphyton community, the periphyton community decreased in microbial interactions overall when exposed to phosphate or nitrate + phosphate. Nitrate microbial networks also demonstrated increased cross-kingdom interactions as it was the only microbial network with a fungal hub representative. The increase in interactions furthers the potential that the periphyton microbiome is nitrogen limited.

A disturbance that targets a hub may cascade through the microbial community (Agler et al., 2016). Here, the control periphyton microbiome was found to have four hub taxa composed of *Desulfomicrobium*, *Erythrobacter*, and *Lacibacter*, all of which were not detected hubs in other nutrient-amended networks. *Desulfomicrobium* are a member of the Desulfovibrionaceae family and are frequently connected to high Hg methylation rates (King et al., 2000; Batten and Scow, 2003). *Clostridium*, the top hub for the nitrate network, is also associated with high levels of Hg methylation. Mercury methylation was highest in control and nitrate samples, suggesting that these well-connected hubs may explain higher methylation rates. Additionally, top hubs for the nitrate + phosphate and phosphate networks were largely dominated by aerobic microorganisms such as Rhizobiaceae, Chitinophagaceae, and Gemmatimonadaceae, suggesting that the reduction of Hg methylation potential may be from the loss of anaerobic microorganisms commonly associated with Hg methylation (Gilmour et al., 2013; Podar et al., 2015; McDaniel et al., 2020). The loss of anaerobic microorganisms may indicate a shift of an anaerobic environment to an aerobic environment after nitrate + phosphate and phosphate treatments, but it is not clear if the hub change is due to altered redox status or if the hub changes disrupted the redox dynamics of periphyton. The lower Hg methylation rates we observed in phosphate and nitrate + phosphate treatments may represent disruptions of the hubs found in control samples and merit further exploration.

Nitrate inhibition of MMHg production has previously been demonstrated in oxygen-limited systems (Todorova et al., 2009), but in our study, we found nitrate-amended periphyton MMHg production to be comparable to control samples. Biofilms can contain steep gradients of oxygen on the microscopic scale (Jørgensen et al., 1979; Revsbech et al., 1983) with MMHg generated by SRB and methanogenic bacteria in the anoxic niches within the biofilm. In our study, it is possible that the nitrate addition is utilized by the surrounding microbiome in oxic niches, but future research is needed to determine the impact of nitrate addition on MMHg methylation across the periphyton biofilm oxygen gradient. Interestingly, when nitrate was paired with phosphorus, we found that MMHg methylation decreased similarly to the phosphorus treatment. This difference could be attributed to the reduction of algal biomass in nitrate and nitrate + phosphate treatments. Periphyton algal abundance has previously been linked to methylation rates (Lanza et al., 2017) and phosphorus can be toxic to periphyton algal communities (Beck and Hall, 2018).

SRB are important to both Hg methylation and demethylation (Achá et al., 2011). In our study, we found a strong relationship between the SRB family *Desulfovibrionaceae* and both methylation and demethylation rates. Previous research found that Hg methylation and demethylation were performed by different SRB taxa (King et al., 2000; Hamelin et al., 2011). Interestingly, in our study, nutrient exposures altered Hg methylation potential but did not change demethylation potential, which may suggest that SRB underlying Hg methylation are sensitive to nutrient alterations while demethylating SRB are not. However, surface water controls showed substantial demethylation, indicating that much, but not all, of the demethylation was photodemethylation, making it difficult to determine the relationship between SRB and demethylation measurements.

Periphyton has been shown to aid in the bioremediation of heavy metals (Serra et al., 2009; Bradac et al., 2010; Bere et al., 2012), the degradation of organic compounds (Azim et al., 2003), and heavy metal removal (Wu et al., 2012). To date, most studies have used natural periphyton communities (Bowes et al., 2012; Baxter et al., 2013) with varying success. Given the importance of hub abundance to microbiome function demonstrated here and in other microbiomes, higher success may be achieved through a synthetic microbiome approach (Toju et al., 2020) utilizing microbial hubs identified in the microbial network analyses to maximize periphyton functionality.

In summary, our study found that the Hg-methylating bacterial community changed with nutrient amendments and season. These changes correlated with specific bacterial, archaeal, and fungal members of the corresponding periphyton microbiome. Together, this suggests that monitoring of the entire microbiome in addition to the Hg-methylating community is important in evaluating Hg methylation-demethylation dynamics in natural environments.

## Data Availability Statement

The datasets presented in this study can be found in online repositories. The names of the repository/repositories and accession number(s) can be found at: https://www.ncbi.nlm.nih.gov/, PRJNA688286.

## Author Contributions

GS, SB, and MC contributed to the conception, design, and implementation of the study. GS performed functional measurements and mercury methylation and demethylation assays. KM ran transient availability kinetics models. AC prepared amplicon libraries, analyzed microbiomes, performed microbial network analyses, and performed all statistical analyses. AW and DK extracted microbiomes and generated sequencing data. AC wrote the manuscript with assistance from GS, MC, CG, DE, RW, DK, AW, KM, and SB. All authors contributed to the article and approved the submitted version.

### Conflict of Interest

The authors declare that the research was conducted in the absence of any commercial or financial relationships that could be construed as a potential conflict of interest.

The reviewer BP declared a shared research group with one of the authors CG at time of review.

## Footnotes

^1^https://ncbi.nlm.nih.gov/bioproject
