# Supplementary material for: Nutrient Exposure Alters Microbial Composition, Structure, and Mercury Methylating Activity in Periphyton in a Contaminated Watershed
Source: Front Microbiol. 2021 Mar 19;12:647861. doi: 10.3389/fmicb.2021.647861 (PMC8017159; doi:10.3389/fmicb.2021.647861)
Supplement: Supplementary file 1 [file Data_Sheet_1.pdf]

Supplementary Material

1 Supplemental figures

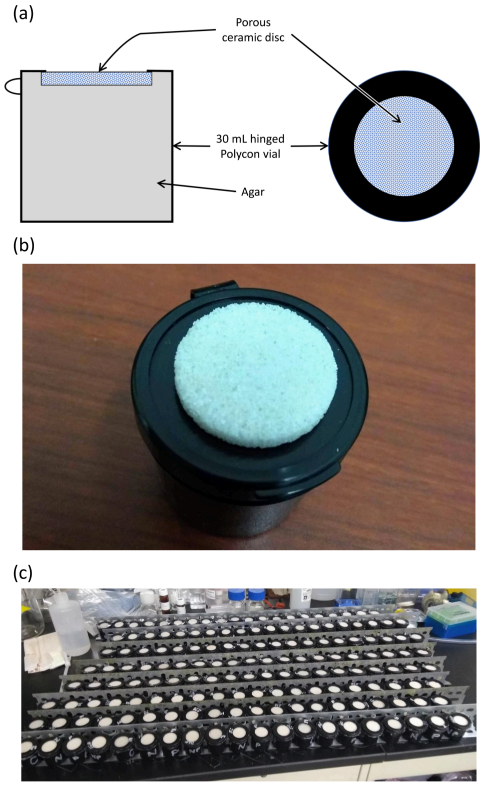

Figure S1: (a) Schematic of NDS cup, (b) substrate disc shown placed on top of NDS cup, and (c) NDS cups attached to angle iron bar just prior to deployment.

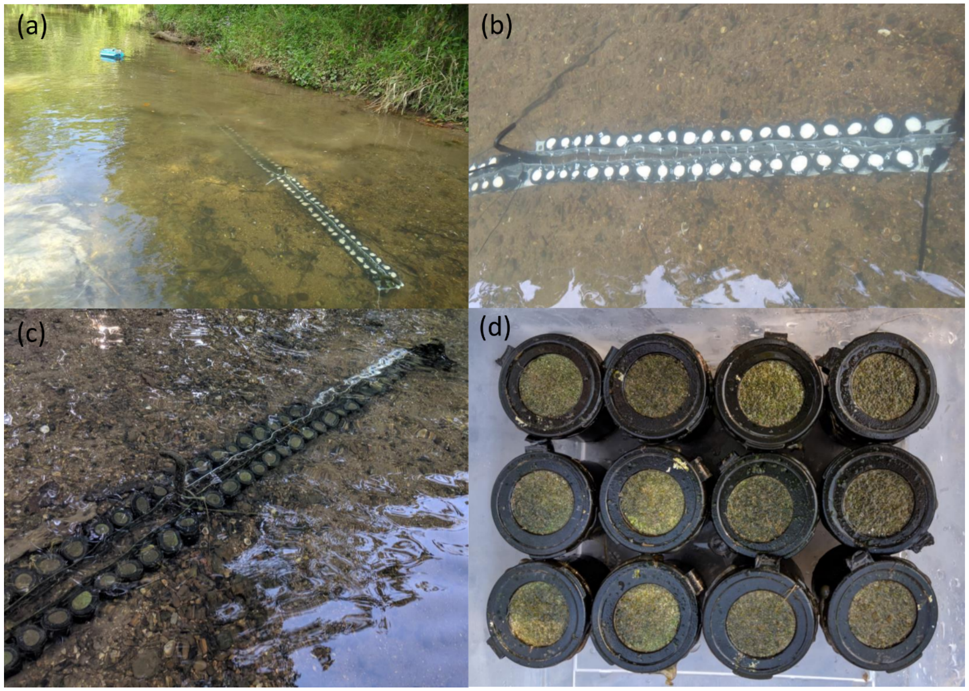

Figure S2: (a) NDS cups in situ on day 1 of deployment with floating light meter shown, (b) NDS cups in situ on day 1 of deployment, (c) NDS cups in situ after one month of deployment, (d) NDS cups after one month of deployment.

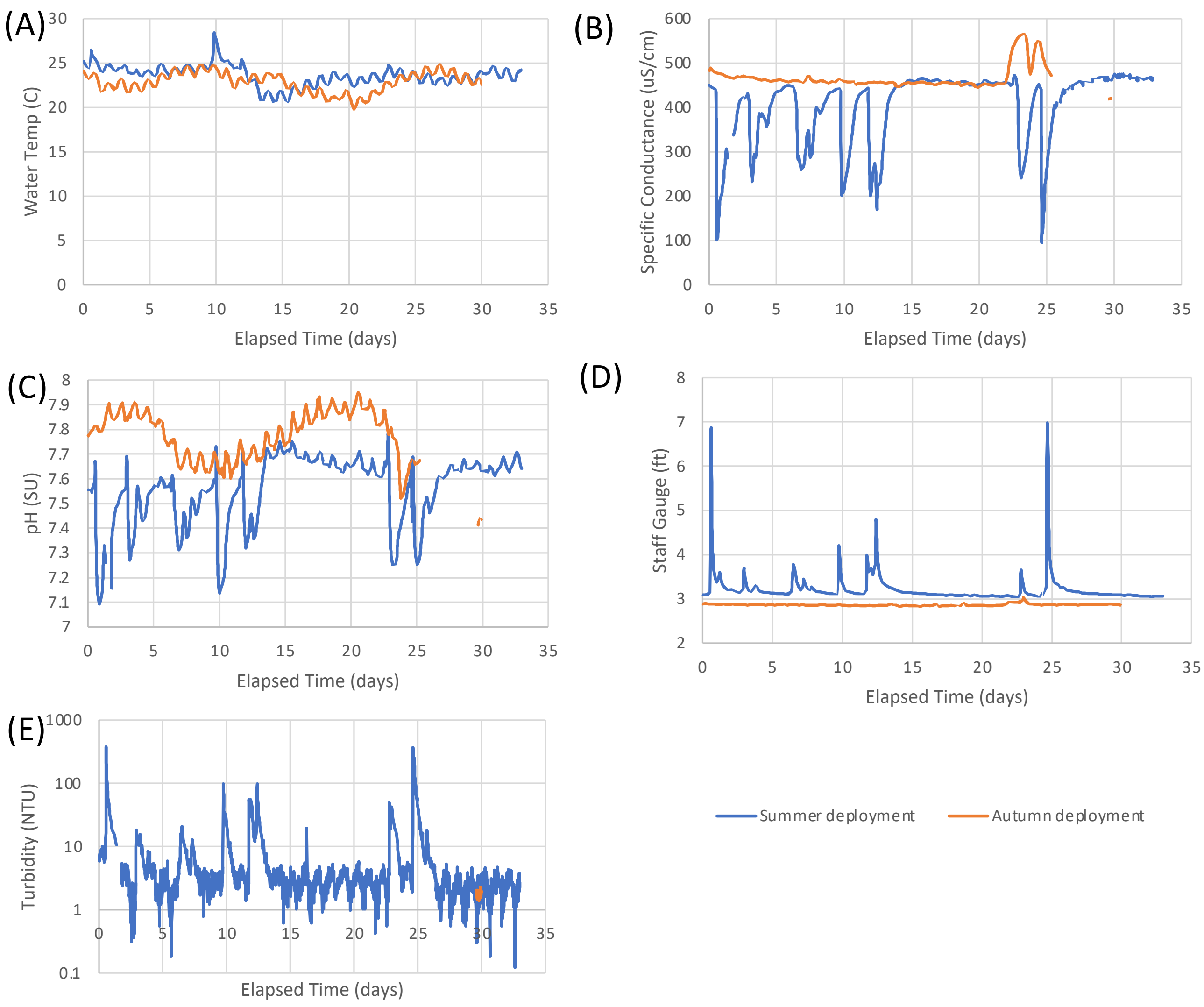

Figure S3: Water measurements (A) water temperature, (B) specific conductivity (C) pH, (D) staff gauge and (E) turbidity were measured every 15 minutes for the duration of the summer and autumn deployments.

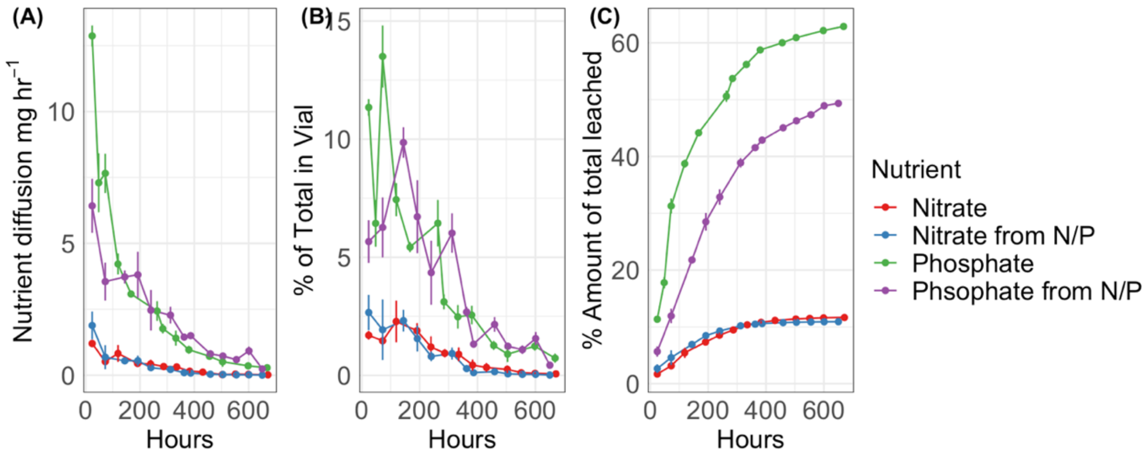

Figure S4: Laboratory-based measurements of the (A) rate of nutrient release from NDS cups over time as measured in the lab, (B) percent of total nutrient content leached at each time point and (C) percent of initial nutrient amount in NDS cups released to solution over time. Data points represent the average of triplicate samples. Error bars represent standard deviation. Each NDS cup was placed in a 1000mL beaker, submerged in 500 mL Milli-Q water, and gently mixed on an orbital shaker at 80 RPM. At each timepoint an aliquot of sample was taken from the beaker water, filtered through a 0.2µM PES syringe filter, and frozen at -20˚C until analysis. The water in the beaker was carefully decanted off, a fresh 500 mL of Milli-Q water was added, and the beaker was returned to the orbital shaker. Timepoints were repeated every 24-72 hours for 1 month. Nitrate in the samples was measured via Ion Chromatography, and phosphate was measured via the Hach Kit molybdenum blue calorimetric method.

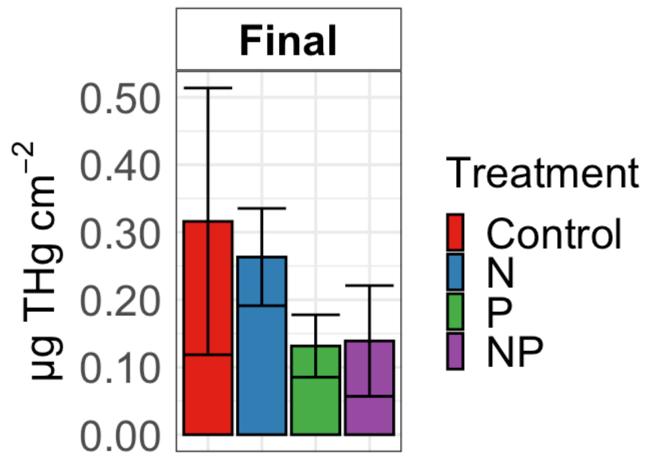

Figure S5: Ambient THg concentration measured at the end of the field experiment of control, nitrate (N), phosphate (P) and nitrate+phosphate (NP) nutrient of periphyton.

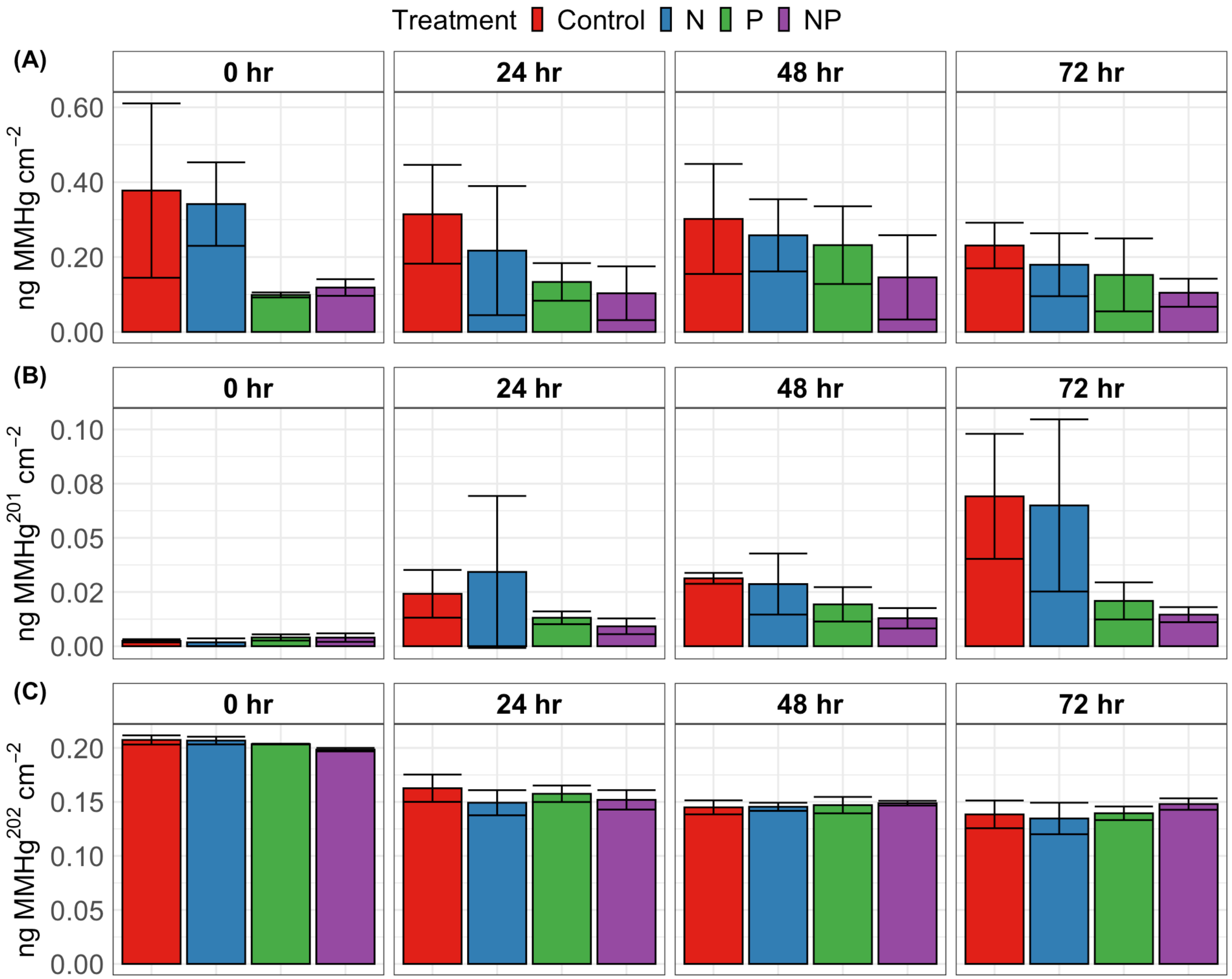

Figure S6: A) ambient MMHg concentration, (B) $MM^{201}Hg$ produced from added inorganic ^{201}Hg, (C) and loss of $MM^{202}Hg$ from added isotope spike over time for control, nitrate (N), phosphate (P) and nitrate+phosphate (NP) nutrient.

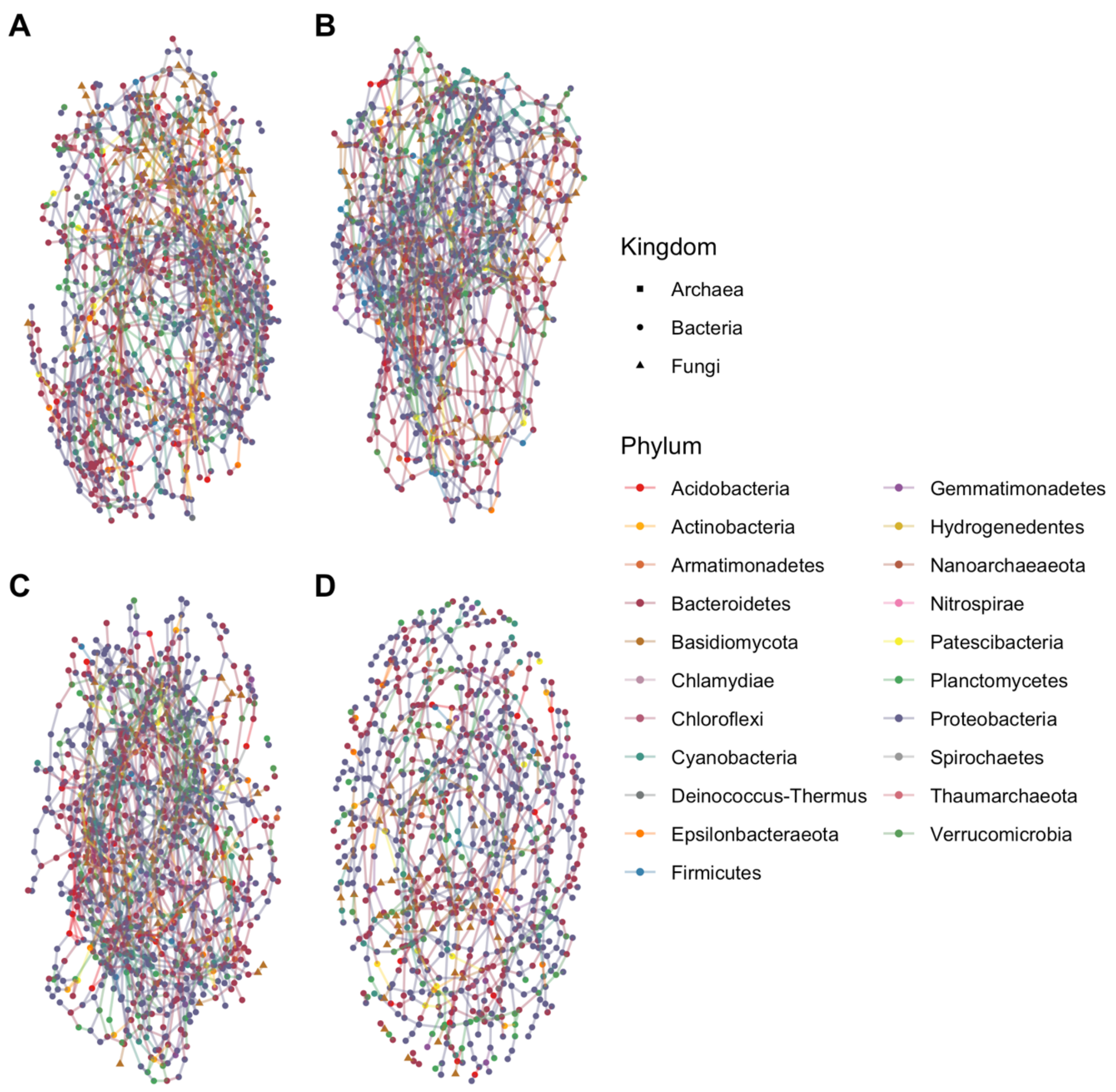

Figure S7: Cross-Domain correlation networks of archaea, bacteria, and fungal taxa calculated with SpiecEasi for control (**A**), nitrate (**B**), phosphate (**C**), and nitrate + phosphate (**D**) nutrient exposure experiments.

2 Supplemental tables

Table S1: Primer sequences for polymerase chain reaction (PCR) amplification.

PRIMER NAME	SEQUENCE	DIRECTION	TARGET	REFERENCE
ITS3NGS1	CATCGATGAAGAACGCAG	Forward	Fungi	White *et al.* 1990[1]
ITS3NGS2	CAACGATGAAGAACGCAG	Forward	Chytridiomycota	Tedersoo *et al.* 2014[2]
ITS3NGS3	CACCGATGAAGAACGCAG	Forward	Sebacinales	Tedersoo *et al.* 2014[2]
ITS3NGS4	CATCGATGAAGAACGTAG	Forward	Glomeromycota	Tedersoo *et al.* 2014[2]
ITS3NGS5	CATCGATGAAGAACGTGG	Forward	Sordariales	Tedersoo *et al.* 2014[2]
ITS3NGS10	CATCGATGAAGAACGCTG	Forward	Stramenopila	Tedersoo *et al.* 2014[2]
ITS4NGR	TCCTSCGCTTATTGATATGC	Reverse	Fungi	White *et al.* 1990[1]
ARCH-ITS4	TCCTCGCCTTATTGATATGC	Reverse	Archaearhizomycetes	Cregger *et al.* 2018[3]
515F	GTGCCAGCMGCCGCGGTAA	Forward	Bacteria/Archaea	Lane *et al.* 1985[4]

515F_f1C	GTGCCAGCMGCWGCGGTAA	Forward	Cloroflexi	Shakya *et al.* 2013[5]
515F_f1TM7	GTGCCAGCMGCCGCGGTCA	Forward	TM7	Shakya *et al.* 2013[5]
515F_f4Arc	GTGKCAGCMGCCGCGGTAA	Forward	Archaea	Shakya *et al.* 2013[5]
806R	GGACTACHVGGGTWTCTAAT	Reverse	Bacteria/Archaea	Lane *et al.* 1985[4]
ORNL-HgcAB-uni-F	AAYGTCTGGTGYGCNGCVGG	Forward	Hg-Methylating bacteria	Gionfriddo *et al.* 2020[6]
ORNL-HgcAB-uni-32R	CAGGCNCCGCAYTCSATRCA	Reverse	Hg-Methylating bacteria	Gionfriddo *et al.* 2020[6]

[1]**Lane DJ, Pace B, Olsen GJ, Stahl DA, Sogin ML, Pace NR**. **1985**. Rapid determination of 16S ribosomal RNA sequences for phylogenetic analyses. *Proceedings of the National Academy of Sciences* **82**: 6955–6959.

[2]**Tedersoo L, Bahram M, Põlme S, Kõljalg U, Yorou NS, Wijesundera R, Ruiz LV, Vasco-Palacios AM, Thu PQ, Suija A, *et al.* 2014**. Global diversity and geography of soil fungi. *Science* **346**.

[3] **Cregger MA, Veach AM, Yang ZK, Crouch MJ, Vilgalys R, Tuskan GA, Schadt CW**. **2018**. The Populus holobiont: dissecting the effects of plant niches and genotype on the microbiome. *Microbiome* **6**: 31.

[4]**White TJ, Bruns T, Lee S, Taylor J. 1990**. Amplification and direct sequencing of fungal ribosomal RNA genes for phylogenetics. In: Innis MA, Gelfand DH, Sninsky JJ, White TJ (eds) PCR Protocols: a Guide to Methods and Applications, pp. 315-322. Academic Press, New York.

[5] **Shakya M, Gottel N, Castro H, Yang ZK, Gunter L, Labbé J, Muchero W, Bonito G, Vilgalys R, Tuskan G, *et al.* 2013**. A Multifactor Analysis of Fungal and Bacterial Community Structure in the Root Microbiome of Mature Populus deltoides Trees. *PLoS ONE* **8**.

[5] **Gionfriddo, C. M., Wymore, A. M., Jones, D. S., Wilpiszeski, R. L., Lynes, M. M., Christensen, G. A., et al. (2020).** An Improved hgcAB Primer Set and Direct High-Throughput Sequencing Expand Hg-Methylator Diversity in Nature. Frontiers in Microbiology **11**.

Table S2: Results of Pearson correlation of periphyton functional measurements and microbial diversity for bacteria (16S rRNA), fungi (ITS2), and mercury methylating microorganisms (hgcAB). Chlorophyll *a*, *b*, *c*, and methylation and demethylation potentials of periphyton exposed to nitrate, phosphate and nitrate+phosphate in summer and autumn 2019. Mercury methylation and demethylation potentials refer to the fitted rate constant from the transient availability model. Significance is denoted with an asterisk. *p<0.05

	**16S rRNA**	**ITS2**	**hgcAB**
**Hg**	0.06	0.12	-0.04
**chlorophyll *a***	-0.12	-0.04	0.08
**chlorophyll *b***	-0.35	-0.20	0.25
**chlorophyll *c***	-0.22	-0.05	0.16
**CR**	-0.19	0.36	-0.35
**GPP**	0.36	-0.10	-0.07
**NCM**	*0.40	-0.08	-0.07
**Methylation Potential**	-0.32	0.41	*-0.58
**Demethylation Potential**	-0.17	0.37	-0.50

Table S3: Pearson correlation was used to test the relationship of 16S rRNA bacterial families and functional measurements: Chlorophyll a, *b*, *c*. Significance is denoted with an asterisk. *p<0.05, **p<0.01, ***p<0.001

**Bacterial Family**	**chlorophyll *a***	**chlorophyll *b***	**chlorophyll *c***
0319-6G20	-0.02	*-0.42	-0.37
A0839	**-0.56	-0.19	-0.19
A4b	-0.35	**-0.53	-0.02
AB1	-0.18	**0.56	-0.01
Acetobacteraceae	-0.26	*-0.51	-0.26
Acetobacterales Incertae Sedis	**0.53	0.37	*0.4
Acidaminococcaceae	*0.41	0.17	0.26
Acidobacteria bacterium SCN 69-37	0	-0.13	-0.3
Amoebophilaceae	-0.15	**-0.62	-0.3
Anaplasmataceae	0.04	-0.04	-0.13
Archangiaceae	-0.38	*-0.49	-0.04

Arcobacteraceae	**0.61	0.02	*0.41
Armatimonadaceae	**-0.59	0.16	**-0.57
Azospirillaceae	-0.35	-0.05	-0.37
BIrii41	-0.37	-0.2	*-0.46
BSV26	-0.32	*-0.47	0.01
Bacteroidaceae	-0.02	0.38	*0.45
Bacteroidetes BD2-2	-0.06	**-0.61	0.12
Bacteroidetes vadinHA17	-0.27	**-0.6	0.03
Balneolaceae	-0.21	*-0.44	-0.31
Beggiatoaceae	0	-0.23	0.33
Burkholderiaceae	0.14	**0.56	-0.01
Caedibacteraceae	*-0.51	0.2	-0.29
Candidatus Lloydbacteria	-0.33	-0.09	*-0.41
Caulobacteraceae	-0.17	***0.64	-0.2

Chitinophagaceae	-0.17	*0.42	-0.23
Chlorobiaceae	-0.32	*-0.43	-0.01
Chromatiaceae	-0.16	**-0.59	0.09
Chroococcidiopsaceae	-0.02	0.37	0.22
Clostridiaceae 1	**0.55	0.06	0.31
Crocinitomicaceae	**0.53	-0.27	0.15
Cyanobacteriaceae	-0.38	-0.38	*-0.51
Cyanobiaceae	-0.34	**-0.57	-0.32
Cyclobacteriaceae	**0.59	*-0.45	0.29
Cytophagaceae	**-0.53	*-0.44	**-0.61
DEV007	*-0.44	-0.33	*-0.51
Desulfobacteraceae	*-0.42	**-0.55	-0.36
Desulfobulbaceae	-0.08	**-0.58	0.24
Desulfomicrobiaceae	0.09	*-0.49	-0.04

Desulfovibrionaceae	0.28	-0.11	*0.49
Devosiaceae	0.09	***0.77	-0.08
Diplorickettsiaceae	-0.23	-0.34	-0.36
Dysgonomonadaceae	*0.42	-0.15	*0.45
Enterobacteriaceae	0	**0.57	0.11
Entomoplasmatales Incertae Sedis	0.04	-0.04	-0.13
FFCH16767	-0.15	**-0.54	-0.05
Family XII	-0.11	*-0.47	0.14
Family XIII	0.11	0.29	0.24
Fimbriimonadaceae	*-0.42	-0.12	-0.09
Flavobacteriaceae	-0.28	**0.52	*-0.51
Fusobacteriaceae	-0.21	-0.29	*-0.4
Gemmataceae	*-0.49	*-0.44	*-0.43
Gemmatimonadaceae	-0.15	-0.02	-0.04

Geobacteraceae	-0.08	**-0.55	0.27
Haliangiaceae	**-0.62	-0.32	-0.18
Halieaceae	-0.31	***-0.66	-0.32
Halomonadaceae	*-0.46	-0.17	**-0.53
Herpetosiphonaceae	*0.5	0.36	0.34
Holophagaceae	-0.02	*-0.49	0.17
Hydrogenedensaceae	0.25	-0.2	*0.44
Hymenobacteraceae	0.22	**-0.58	0.24
Hyphomonadaceae	-0.3	-0.21	-0.12
Iamiaceae	-0.29	*-0.46	-0.16
Ignavibacteriaceae	-0.18	**-0.54	-0.06
Ilumatobacteraceae	-0.39	*-0.43	-0.39
Intrasporangiaceae	-0.04	***0.81	0.21
JG30-KF-CM45	0.05	*0.45	0.29

Kaistiaceae	*-0.43	0.01	***-0.68
Kineosporiaceae	-0.06	-0.13	-0.32
Latescibacteraceae	*-0.45	-0.37	-0.24
Lentimicrobiaceae	-0.3	*-0.48	0.11
Leptolyngbyaceae	0.12	-0.33	-0.15
Leptospiraceae	-0.31	*-0.42	-0.23
LiUU-11-161	-0.18	*-0.5	*-0.41
Limnotrichaceae	-0.1	0.38	-0.11
M2PB4-65 termite group	-0.04	**-0.54	0.18
Marinilabiliaceae	*0.41	*-0.42	0.36
Methylomirabilaceae	-0.18	**-0.52	-0.11
Methylophilaceae	***0.63	0.26	0.4
Micavibrionaceae	-0.13	*-0.49	-0.32
Microbacteriaceae	-0.22	-0.14	*-0.45

Micrococcaceae	-0.12	-0.07	-0.3
Microcystaceae	-0.31	-0.2	*-0.5
Micropepsaceae	-0.15	**0.58	0.15
Microscillaceae	-0.28	*-0.46	*-0.44
Microtrichaceae	-0.3	-0.23	**-0.58
Midichloriaceae	-0.22	-0.08	-0.16
Moraxellaceae	-0.01	0.34	*0.41
NS11-12 marine group	0.06	*-0.46	-0.28
Nannocystaceae	-0.09	*-0.48	-0.07
Neisseriaceae	*-0.46	-0.03	**-0.59
Nitrosomonadaceae	*-0.44	*-0.43	-0.18
Nitrosopumilaceae	*-0.45	**-0.58	-0.19
Nitrospiraceae	*-0.41	**-0.56	-0.28
Nostocaceae	-0.1	0.12	0.11

Oligoflexaceae	-0.28	0.35	*-0.45
Paenibacillaceae	-0.17	*0.47	-0.29
Paludibacteraceae	0.04	*0.43	0.31
Paracaedibacteraceae	-0.26	*-0.4	-0.22
Parachlamydiaceae	-0.04	*-0.5	-0.26
Parcubacteria	0.05	-0.05	-0.19
Pedosphaeraceae	*-0.5	-0.31	-0.38
Peptococcaceae	**0.57	-0.15	0.27
Peptostreptococcaceae	-0.26	**-0.53	0.04
Phycisphaeraceae	*-0.45	-0.21	*-0.49
Pirellulaceae	-0.35	**-0.6	-0.26
Piscirickettsiaceae	-0.26	*-0.5	-0.22
Prolixibacteraceae	-0.13	**-0.56	0.2
Propionibacteriaceae	0.08	*0.41	*0.41

Pseudohongiellaceae	-0.2	-0.31	-0.32
Pseudomonadaceae	*0.43	0.18	0.36
Puniceicoccaceae	-0.38	-0.26	**-0.59
Rhizobiaceae	-0.06	***0.77	0.12
Rhodanobacteraceae	-0.12	**0.52	0.1
Rhodobacteraceae	0.12	*-0.46	0.24
Rhodocyclaceae	*0.43	**-0.61	*0.49
Rhodothermaceae	0.04	-0.04	-0.13
Rikenellaceae	0.24	-0.35	0.38
Rubinisphaeraceae	-0.28	**-0.52	-0.13
Rubritaleaceae	-0.27	*-0.45	-0.29
Ruminococcaceae	0.09	0.25	0.25
SB-5	*-0.41	**-0.61	-0.18
SC-I-84	*-0.43	*-0.43	-0.07

SM2D12	*-0.5	-0.05	**-0.54
SS1-B-06-26	-0.14	*-0.42	-0.2
Sandaracinaceae	*-0.48	-0.38	**-0.58
Saprospiraceae	-0.31	**-0.62	-0.07
Schlesneriaceae	-0.18	*-0.43	-0.21
Shewanellaceae	***0.69	-0.02	*0.42
Solibacteraceae (Subgroup 3)	**-0.52	-0.26	-0.22
Spongiibacteraceae	-0.24	**-0.61	-0.19
Stappiaceae	0.11	*-0.48	-0.12
Steroidobacteraceae	-0.36	*-0.47	-0.1
Streptomycetaceae	-0.3	0.28	*-0.45
TRA3-20	*-0.44	-0.29	*-0.41
Tannerellaceae	0.24	0.28	0.2
Thermosynechococcaceae	0.03	-0.19	-0.02

Unclassified	0	**-0.62	0.26
Unknown Family	*-0.43	0.23	-0.1
Veillonellaceae	*0.46	0.15	0.34
Vermiphilaceae	-0.19	-0.14	-0.38
WD2101 soil group	*-0.46	-0.13	-0.15
Weeksellaceae	0.19	**0.6	0.27
Woeseiaceae	-0.3	*-0.5	-0.39
Xanthobacteraceae	*-0.49	0.3	*-0.5
Xanthomonadaceae	-0.04	0.35	-0.3
Xenococcaceae	-0.06	*-0.47	-0.2
bacterium LWQ8	-0.3	-0.11	*-0.46
env.OPS 17	-0.29	-0.4	*-0.45
marine metagenome	0.03	-0.04	-0.13
mle1-27	-0.21	0	-0.15

uncultured Acidobacterium sp.	-0.27	*-0.45	0.11
uncultured Bacteroidetes	-0.37	-0.19	*-0.44
uncultured Chlorobi bacterium	-0.16	-0.25	*-0.48
uncultured Latescibacteria bacterium	-0.32	-0.1	*-0.41
uncultured Oceanibaculum sp.	*-0.43	-0.37	**-0.59
uncultured Planctomycetales bacterium	*-0.49	*-0.42	-0.29
uncultured Rhodospirillaceae bacterium	0.04	-0.04	-0.13
uncultured bacterium	*-0.44	*-0.5	-0.22
uncultured bacterium SBR2013	0.18	*0.42	0.33
uncultured candidate division SR1 bacterium	-0.13	0.4	-0.03
uncultured cyanobacterium	-0.03	-0.03	-0.23
uncultured delta proteobacterium	-0.09	-0.11	-0.29

uncultured gamma proteobacterium	*-0.43	-0.24	***-0.67
uncultured marine bacterium	0.2	0.26	0.23
uncultured microorganism	*-0.42	***-0.68	*-0.43
uncultured sludge bacterium A12b	-0.27	-0.34	*-0.51
uncultured soil bacterium	*-0.48	-0.23	*-0.5

Table S4: Pearson correlation was used to test the relationship of 16S rRNA bacterial families and mercury methylation and demethylation potential. Mercury methylation and demethylation potentials refer to the fitted rate constant from the transient availability model. Significance is denoted with an asterisk. *p<0.05, **p<0.01, ***p<0.001

**Family**	**Methylation**	**Demethylation**
37-13	*-0.58	-0.48
A0839	**-0.82	*-0.64
Aeromonadaceae	0.49	*0.62

Alteromonadaceae	*0.6	0.34
Arcobacteraceae	*0.67	*0.65
Azospirillaceae	*-0.65	-0.51
Blastocatellaceae	*-0.61	*-0.63
Burkholderiaceae	0.57	*0.63
Caedibacteraceae	*-0.6	-0.44
Cellvibrionaceae	0.16	0.11
Chloracidobacteriaceae	*-0.66	-0.53
Clostridiaceae 1	0.5	*0.59
Cyanobacteriaceae	*-0.66	-0.45
Cyanobiaceae	*-0.58	-0.43
Cyclobacteriaceae	**0.72	**0.73
Desulfarculaceae	-0.08	-0.08
Desulfovibrionaceae	*0.6	*0.59

Dongiaceae	**-0.73	*-0.64
Dysgonomonadaceae	*0.58	0.52
Eubacteriaceae	*0.65	0.54
Family XIII	*0.59	0.51
Fimbriimonadaceae	**-0.8	*-0.65
Flavobacteriaceae	**-0.71	*-0.6
Francisellaceae	0.45	0.36
Gemmataceae	*-0.65	-0.49
Gemmatimonadaceae	**-0.76	-0.52
Gimesiaceae	0.04	0.26
Gracilibacteria bacterium canine oral taxon 394	*0.58	0.38
Hydrogenedensaceae	0.43	0.48
Hyphomonadaceae	***-0.91	**-0.78
Kaistiaceae	***-0.88	-0.58

Lacibacterium aquatile	**-0.72	-0.56
Leptospiraceae	*-0.67	-0.41
Marinilabiliaceae	*0.66	0.55
Methylophilaceae	*0.67	**0.78
Microbacteriaceae	*-0.58	-0.55
Microcystaceae	*-0.62	-0.52
Microscillaceae	*-0.65	-0.41
Midichloriaceae	*-0.59	*-0.65
NS11-12 marine group	*-0.69	-0.44
Oligoflexaceae	**-0.79	**-0.72
PHOS-HE36	0.37	0.54
Paludibacteraceae	*0.63	0.32
Pedosphaeraceae	*-0.71	-0.42
Peptostreptococcaceae	0.24	0.33

Phycisphaeraceae	**-0.78	-0.5
Pseudanabaenaceae	*-0.59	-0.4
Pseudomonadaceae	0.52	*0.59
Reyranellaceae	**-0.71	-0.36
Rhizobiales Incertae Sedis	*-0.71	*-0.68
Rhodocyclaceae	***0.85	*0.66
Rikenellaceae	**0.78	*0.59
Roseiflexaceae	**-0.73	-0.53
Rubritaleaceae	*-0.59	-0.51
SM2D12	*-0.69	-0.49
Shewanellaceae	0.55	*0.65
Solibacteraceae (Subgroup 3)	*-0.58	-0.26
TRA3-20	**-0.77	-0.51
Tannerellaceae	0.49	*0.58

Tepidisphaeraceae	*-0.64	-0.53
Thermaceae	-0.46	*-0.66
VHS-B3-70	-0.22	-0.19
Veillonellaceae	*0.58	*0.63
Vermiphilaceae	*-0.67	**-0.72
WD2101 soil group	**-0.71	*-0.6
Weeksellaceae	*0.58	*0.61
Xanthobacteraceae	***-0.9	*-0.61
bacterium enrichment culture clone Anammox_49	-0.54	*-0.66
env.OPS 17	*-0.66	-0.41
uncultured Acidobacterium sp.	0.31	0.38
uncultured Bacteroidetes/Chlorobi group bacterium	*-0.69	-0.47
uncultured Chlorobi bacterium	*-0.6	-0.57

uncultured Oceanibaculum sp.	*-0.68	-0.53
uncultured candidate division SBR1093 bacterium	0.13	0.08
uncultured delta proteobacterium	-0.14	-0.21
uncultured gamma proteobacterium	**-0.81	*-0.63
uncultured microorganism	**-0.79	*-0.62
uncultured proteobacterium	*-0.66	-0.46
uncultured sludge bacterium A12b	*-0.71	*-0.59
uncultured sulfur-oxidizing symbiont bacterium	-0.09	0.04

Table S5: Pearson correlation was used to test the relationship of mercury methylating families and functional measurements. Significance is denoted with an asterisk. *p<0.05, **p<0.01, ***p<0.001

**Family**	**chlorophyll *a***	**chlorophyll *b***	**chlorophyll *c***
Clostridiaceae	0.02	0.38	-0.22

Desulfarculaceae	**0.61	0.19	0.16
Desulfobacteraceae	0.02	0.37	-0.1
Desulfobulbaceae	0.33	0.29	*0.46
Desulfohalobiaceae	0.11	0.12	-0.02
Desulfomicrobiaceae	0.08	-0.08	-0.24
Geobacteraceae	0.08	*0.43	-0.05
Methanoregulaceae	0.11	**0.52	-0.07
Methanosarcinaceae	0.19	0.39	0.1
Peptococcaceae	-0.08	0.31	0.18
Spirochaetaceae	0.18	0.05	0.04
Syntrophobacteraceae	0.22	0.03	-0.04
Thermococcaceae	0.39	*0.46	0.33

Table S6: Pearson correlation was used to test the relationship of mercury methylating families and mercury methylation and demethylation potential. Significance is denoted with an asterisk. *p<0.05, **p<0.01, ***p<0.001

Family	Methylation	Demethylation
Clostridiaceae	*-0.69	***-0.85
Desulfobacteraceae	*-0.62	*-0.7
Desulfohalobiaceae	-0.46	*-0.61
Desulfomicrobiaceae	0.57	**-0.78
Desulfovibrionaceae	**0.77	*-0.65
Geobacteraceae	-0.52	*-0.69
Methanoregulaceae	*-0.62	**-0.73
Methanosarcinaceae	-0.5	*-0.68
Nitrospinaceae	*-0.6	**-0.82
Syntrophobacteraceae	0.5	*-0.59

Table S7: Cross-domain hubs from microbial networks for all treatments

**Kingdom**	**Phylum**	**Class**	**Order**	**Family**	**Genus**
Bacteria	Proteobacteria	Deltaproteobacteria	Desulfovibrionales	Desulfomicrobiaceae	Desulfomicrobium
Bacteria	Proteobacteria	Alphaproteobacteria	Sphingomonadales	Sphingomonadaceae	Erythrobacter
Bacteria	Bacteroidetes	Bacteroidia	Chitinophagales	Chitinophagaceae	Lacibacter
Bacteria	Bacteroidetes	Bacteroidia	Chitinophagales	Chitinophagaceae	Lacibacter
Bacteria	Bacteroidetes	Bacteroidia	Flavobacteriales	Flavobacteriaceae	Flavobacterium
Bacteria	Bacteroidetes	Bacteroidia	Flavobacteriales	Flavobacteriaceae	Flavobacterium
Bacteria	Bacteroidetes	Bacteroidia	Flavobacteriales	Flavobacteriaceae	Flavobacterium
Bacteria	Bacteroidetes	Bacteroidia	Flavobacteriales	Crocinitomicaceae	Fluviicola
Bacteria	Proteobacteria	Gammaproteobacteria	Betaproteobacteriales	Burkholderiaceae	Hydrogenophaga
Bacteria	Proteobacteria	Gammaproteobacteria	Betaproteobacteriales	Burkholderiaceae	Hydrogenophaga
Bacteria	Verrucomicrobia	Verrucomicrobiae	Verrucomicrobiales	Rubritaleaceae	Luteolibacter
Bacteria	Hydrogenedentes	Hydrogenedentia	Hydrogenedentiales	Hydrogenedensaceae	metagenome

Bacteria	Proteobacteria	Gammaproteobacteria	R7C24	metagenome	metagenome
Bacteria	Cyanobacteria	Oxyphotobacteria	Nostocales	Microcystaceae	Microcystis PCC-7914
Fungi	Basidiomycota	NA	NA	NA	NA
Bacteria	Cyanobacteria	Oxyphotobacteria	Leptolyngbyales	Leptolyngbyaceae	NA
Bacteria	Cyanobacteria	Melainabacteria	Gastranaerophilales	NA	NA
Bacteria	Firmicutes	Clostridia	Clostridiales	Clostridiaceae	Clostridium
Bacteria	Proteobacteria	Gammaproteobacteria	Betaproteobacteriales	Rhodocyclaceae	NA
Bacteria	Proteobacteria	Alphaproteobacteria	Sphingomonadales	Sphingomonadaceae	Novosphingobium
Bacteria	Bacteroidetes	Bacteroidia	Cytophagales	Microscillaceae	OLB12
Bacteria	Verrucomicrobia	Verrucomicrobiae	Verrucomicrobiales	Verrucomicrobiaceae	Prosthecobacter
Bacteria	Proteobacteria	Alphaproteobacteria	Reyranellales	Reyranellaceae	Reyranella
Bacteria	Proteobacteria	Alphaproteobacteria	Sphingomonadales	Sphingomonadaceae	Sphingopyxis
Bacteria	Firmicutes	Negativicutes	Selenomonadales	Veillonellaceae	Sporomusa
Bacteria	Proteobacteria	Gammaproteobacteria	Xanthomonadales	Xanthomonadaceae	Stenotrophomonas

Bacteria	Proteobacteria	Gammaproteobacteria	Xanthomonadales	Xanthomonadaceae	Stenotrophomonas
Bacteria	Bacteroidetes	Bacteroidia	Chitinophagales	Chitinophagaceae	Terrimonas
Bacteria	Proteobacteria	Gammaproteobacteria	Betaproteobacteriales	Rhodocyclaceae	Thauera
Bacteria	Cyanobacteria	Oxyphotobacteria	Leptolyngbyales	Leptolyngbyaceae	uncultured
Bacteria	Verrucomicrobia	Verrucomicrobiae	Verrucomicrobiales	Verrucomicrobiaceae	uncultured
Bacteria	Bacteroidetes	Bacteroidia	Bacteroidales	Rikenellaceae	uncultured bacterium
Bacteria	Bacteroidetes	Bacteroidia	Bacteroidales	Prolixibacteraceae	WCHB1-32
Bacteria	Proteobacteria	Gammaproteobacteria	Betaproteobacteriales	Rhodocyclaceae	Azospira
Bacteria	Proteobacteria	Deltaproteobacteria	Bdellovibrionales	Bdellovibrionaceae	Bdellovibrio
Bacteria	Acidobacteria	Subgroup 6	NA	NA	NA
Bacteria	Bacteroidetes	Bacteroidia	Sphingobacteriales	Sphingomonadaceae	NA
Bacteria	Proteobacteria	Alphaproteobacteria	Acetobacterales	Acetobacterales Incertae Sedis	NA
Bacteria	Verrucomicrobia	Verrucomicrobiae	Pedosphaerales	Pedosphaeraceae	NA

Bacteria	Bacteroidetes	Bacteroidia	Chitinophagales	Chitinophagaceae	Sediminibacterium
Bacteria	Cyanobacteria	Oxyphotobacteria	Leptolyngbyales	Leptolyngbyaceae	uncultured
Bacteria	Verrucomicrobia	Verrucomicrobiae	Pedosphaerales	Pedosphaeraceae	uncultured soil bacterium
Bacteria	Proteobacteria	Alphaproteobacteria	Rhizobiales	Rhizobiaceae	Allorhizobium-Neorhizobium-Pararhizob[illegible]
Bacteria	Proteobacteria	Alphaproteobacteria	Rhizobiales	Rhizobiaceae	Allorhizobium-Neorhizobium-Pararhizob[illegible]
Bacteria	Proteobacteria	Gammaproteobacteria	Betaproteobacteriales	Rhodocyclaceae	Azospira
Bacteria	Proteobacteria	Alphaproteobacteria	Rickettsiales	Rickettsiaceae	Candidatus Megaira
Bacteria	Bacteroidetes	Bacteroidia	Chitinophagales	Chitinophagaceae	Chitinophaga
Bacteria	Gemmatimonadetes	Gemmatimonadetes	Gemmatimonadales	Gemmatimonadaceae	Gemmatimonas
Bacteria	Proteobacteria	Gammaproteobacteria	Enterobacteriales	Enterobacteriaceae	NA
Bacteria	Proteobacteria	Alphaproteobacteria	Sphingomonadales	Sphingomonadaceae	Porphyrobacter
Bacteria	Proteobacteria	Alphaproteobacteria	Sphingomonadales	Sphingomonadaceae	Sphingopyxis
Bacteria	Cyanobacteria	Oxyphotobacteria	Leptolyngbyales	Leptolyngbyaceae	uncultured
Bacteria	Proteobacteria	Alphaproteobacteria	Rhizobiales	Rhizobiaceae	uncultured

Bacteria	Bacteroidetes	Bacteroidia	Bacteroidales	Prolixibacteraceae	WCHB1-32
